# A new sighting study for the fixed concentration procedure to allow for gender differences

**DOI:** 10.1177/0960327110370983

**Published:** 2010-03

**Authors:** Nigel Stallard, Charlotte Price, Stuart Creton, Ian Indans, Robert Guest, David Griffiths, Philippa Edwards

**Affiliations:** 1 Warwick Medical School, University of Warwick, Coventry, UK; 2 National Centre for the Replacement, Refinement and Reduction of Animals in Research (NC3Rs), London, UK; 3 Health Directorate, Health and Safety Executive, Bootle, Merseyside, UK; 4 Harlan Laboratories Ltd, Shardlow, UK; 5 Health Protection Agency, Didcot, UK

**Keywords:** acute inhalation toxicity, OECD Test Guidelines, fixed concentration procedure, gender differences

## Abstract

The fixed concentration procedure (FCP) has been proposed as an alternative to the median lethal concentration (LC_50_) test (organisation for economic co-operation and development (OECD) test guideline [TG] 403) for the assessment of acute inhalation toxicity. The FCP tests animals of a single gender (usually females) at a number of fixed concentration levels in a sequential fashion. It begins with a sighting study that precedes the main FCP study and is used to determine the main study starting concentration. In this paper, we propose a modification to the sighting study and suggest that it should be conducted using both male and female animals, rather than just animals of a single gender. Statistical analysis demonstrates that, when females are more sensitive, the new procedure is likely to give the same classification as the original FCP, whereas, if males are more sensitive, the new procedure is much less likely to lead to incorrect classification into a less toxic category. If there is no difference in the LC_50_ for females and males, the new procedure is slightly more likely to classify into a more stringent class than the original FCP. Overall, these results show that the revised sighting study ensures gender differences in sensitivity do not significantly impact on the performance of the FCP, supporting its use as an alternative test method for assessing acute inhalation toxicity.

## Introduction

The current internationally accepted methods for assessing the acute inhalation toxicity of chemicals are the LC_50_ method (described in organisation for economic co-operation and development (OECD) TG 403^1^) and the acute toxic class method (ATC; described in OECD TG 436^2^), both of which require lethality as the endpoint. The fixed concentration procedure (FCP)^3,4^ has been proposed as an alternative method and uses signs of ‘evident toxicity’ as the endpoint. The FCP aims to determine a concentration level that will lead to evident toxicity, where this means clear signs of toxicity indicating that exposure to the next highest concentration would cause severe toxicity requiring euthanasia or death in most animals within 14 days. The FCP provides a refinement in animal welfare terms over the LC_50_ and the ATC methods as it does not require death or severe toxicity as an endpoint. In addition, it uses fewer animals, particularly in comparison with the LC_50_ test.^1^

Since acute toxicity tests are used to assess the potential hazards and risks to human health, the information obtained from the FCP regarding non-lethal signs of toxicity provides additional value. However, a major use of the output of acute toxicity studies is for the purpose of classification and labelling of chemicals, based on their potential hazard. It is therefore important to establish that the proposed FCP protocol is satisfactory for this purpose.

A recent statistical evaluation of the performances of the FCP, the LC_50_ method and ATC method showed that all three methods perform well in the absence of gender differences.^5^ However, performance is affected in all cases when unanticipated gender differences are present. While the effect is relatively minor for the ATC and LC_50_ methods, which test both males and females, the performance of the FCP is substantially worsened. In particular, the statistical evaluation of the FCP when the LC_50_ for males is one-tenth of that for females showed that misclassification into a less toxic category can occur with high probability; in some cases, nearly 100% of the time.

**Figure 1.**
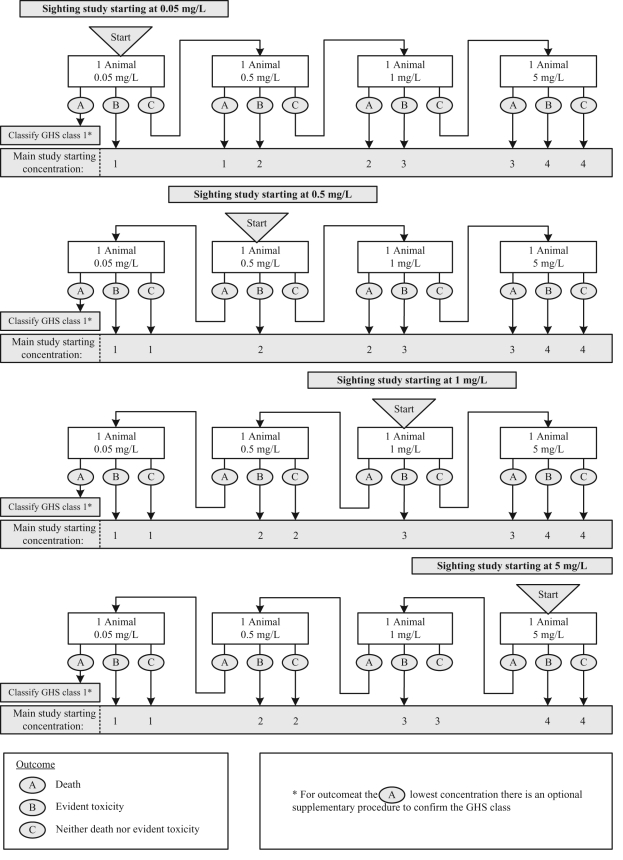
The fixed concentration procedure sighting study for classification of dusts and mists according to the Globally Harmonised Scheme (GHS) classification system.

**Figure 2.**
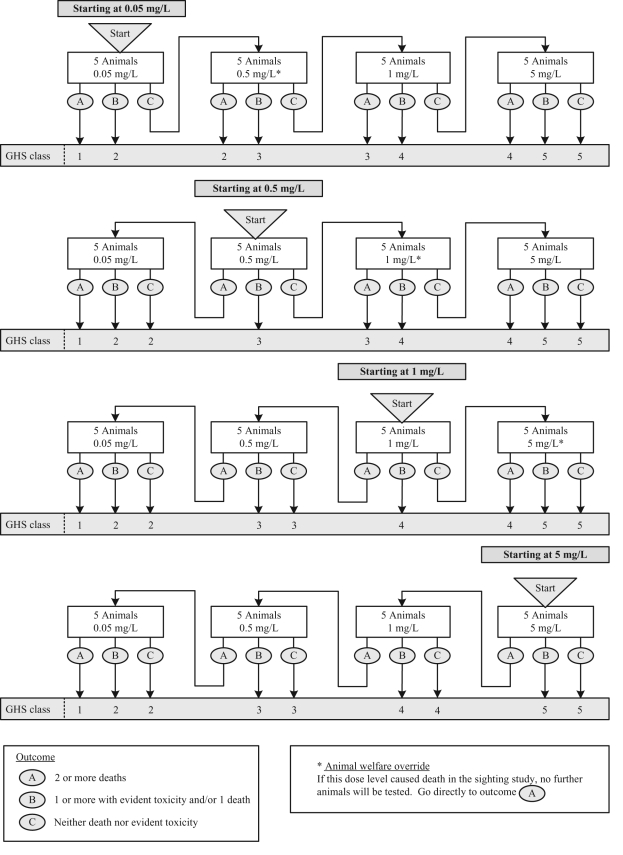
The fixed concentration procedure main study for classification of dusts and mists according to the Globally Harmonised Scheme (GHS) classification system.

The FCP tests animals of a single gender at one or more of four fixed concentration levels in a stepwise manner, and unless it is believed that males are likely to be more sensitive, females are used for testing. The fixed concentrations correspond to the LC_50_ values on the boundaries between the Globally Harmonised Scheme (GHS) acute toxicity classes.^6^ For example, for dusts and mists, testing is conducted at concentrations of 0.05, 0.5, 1 or 5 mg/L. Unless there is reliable prior knowledge about the toxicity of the test substance, for example if a limit test is to be conducted, the study will generally be preceded by a sighting study that is used to determine an appropriate starting concentration for the main study. The sighting study and main study are shown in Figures 1 and 2 , respectively.

If testing in the main study starts at the highest concentration level, such as 5 mg/L for dusts and mists, and no evident toxicity is observed, the substance is regarded as unclassified. In this case, the FCP is effectively a limit test. For substances that do evoke toxicity and thus require full testing, the use of a sighting study to rapidly determine an appropriate starting concentration for the main study, and the use of non-fatal evident toxicity as an endpoint, means that the FCP typically uses considerably fewer animals and leads to fewer deaths than alternative acute inhalation toxicity testing procedures such as those described by TG 403^1^ and TG 436,^2^ as indicated by Price et al.^5^

The use of a single gender in the FCP means that if there are unanticipated gender differences in the sensitivity of the animals to a test substance, and the least sensitive gender is used erroneously, the procedure may lead to an incorrect classification. In a previous analysis of data from the assessment of acute inhalation toxicity for 56 substances using LC_50_ testing, statistically significant gender differences were found in 16 substances (29%).^5^ In the majority of substances where a gender difference was indicated, females were more sensitive, with LC_50_ values for males up to 19 times that for females. However, in some cases, males were more sensitive, with the LC_50_ value for females up to 12 times that for males. These findings demonstrate that the potential for gender differences in sensitivity following inhalation exposure needs to be taken into account in assessing the performance of acute inhalation toxicity test methods.

To address the impaired performance of the FCP when male animals are more sensitive than females, we propose a modification to the sighting study that is used to determine the starting concentration of the main study. It is suggested that the sighting study should be conducted using both male and female animals, as described in detail in the next section. A statistical evaluation of the performance of the revised protocol is presented in the presence and absence of gender differences.

## Methods

### Modification of the FCP to include both genders in the sighting study

**Figure 3.**
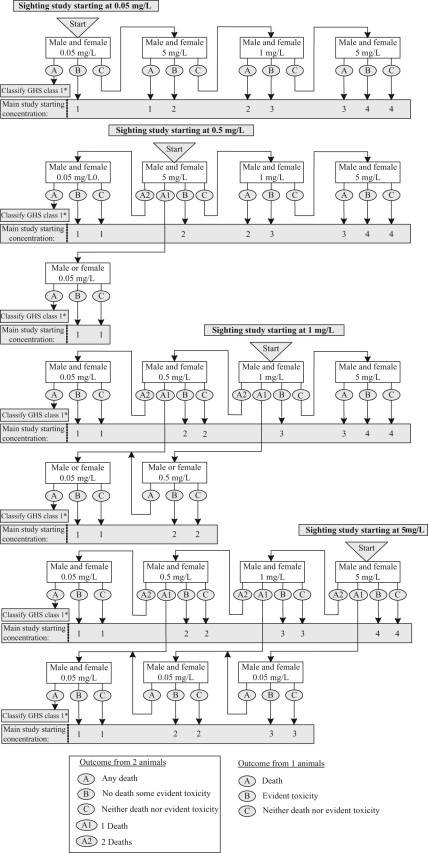
Revised fixed concentration procedure sighting study (dusts and mists).

In order to detect any substantial gender differences in sensitivity to acute inhalation toxicity, as well as selecting an appropriate main study starting concentration, a new sighting study for the FCP is proposed in which both males and females are tested. The new proposed sighting study is shown in Figure 3 . Initially, two animals, one male and one female, are simultaneously exposed at a chosen starting concentration. If both animals demonstrate the same response of death, non-fatal evident toxicity or no effects, the sighting study either stops and leads to a main study conducted in females or continues to test two animals (one male and one female) at the next concentration, exactly as in the original proposed FCP sighting study.

If, at any concentration, a gender difference is indicated, the main study will be conducted using the gender that is shown to be the more sensitive, and the sighting study continues with that gender alone in such a way as to determine an appropriate main study starting concentration. Specifically, if one animal dies and the other survives, the sighting study continues by exposing another animal of the more sensitive gender at the next lower concentration, unless testing at that concentration has already been conducted, in which case the main study starts in the more sensitive gender at the next lower concentration to that at which death occurred. If instead one animal demonstrates evident toxicity and the other shows no toxic effects, the main study starts at that concentration using the gender of the animal in which the evident toxicity was observed.

The main study is conducted using five animals per concentration of the gender indicated by the new sighting study as described above but is otherwise identical to that proposed previously.^3,4^

### Statistical evaluation of the FCP with modified sighting study

Stallard et al.^4^ described a method for the statistical evaluation of the FCP. The method is based on the assumption that both the probability of death and the probability of either death or non-fatal evident toxicity are given by probit concentration-response curves with the same slope. Based on these concentration-response curves, calculations can be performed to obtain the probability of each possible outcome at each of the fixed testing concentrations. This enables the probabilities of classification into each of the toxic classes, together with the average number of animals required and deaths resulting from the testing of hypothetical substances, to be calculated. A gender difference in sensitivity to acute inhalation toxicity may be assumed by including concentration-response curves for males and females with the same slope but different LC_50_ values. Further details are given by Price et al.^5^

Assuming a range of sighting study starting concentrations, the statistical evaluation was conducted for hypothetical dusts and mists with LC_50_ values ranging from 0.01 to 50 mg/L. The ratio of the LC_50_ to the TC_50_, denoted by *R*, was taken to be 5, where the TC_50_ is the concentration expected to cause death or evident toxicity in 50% of the animals. Concentration-response curve slope values of 10 and 4 were investigated. A value of 10 was the median concentration-response curve slope reported by Greiner,^7^ while 4 was the first percentile value, indicating that 1% of substances might have concentration-response curves shallower than this. As the performance of all test methods worsens with shallower concentration-response curves, it is of particular interest to consider this low value. Results were obtained assuming no gender difference or a 10-fold difference in LC_50_ values for males and females.

## Results

**Figure 4.**
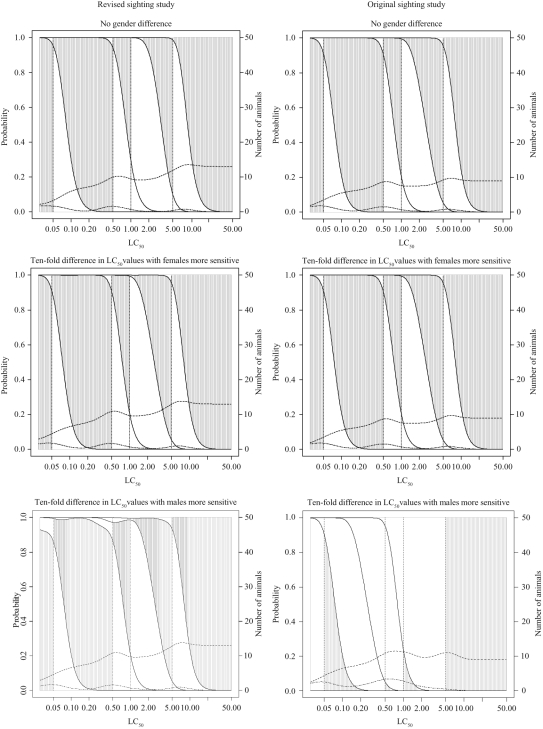
Classification probabilities and expected numbers of animals and deaths for the fixed concentration procedure (FCP) with the new sighting study for dusts and mists with concentration-response curve slope of 4 and *R* (LC_50_/TC_50_) of 5 assuming sighting study starting at 0.05 mg/L. Cumulative probabilities of classification (on left-hand axis scale) into each toxic class for LC_50_ values are shown. The height of the shaded areas gives the probability of correct classification, the height of the area below the shaded area is the probability of classification into too toxic a class and the height of the area above the shaded area is the probability of classification into a class that is not toxic enough. The dashed lines give expected number of animals and deaths (using the scale on the right-hand axis), with the top line indicating the number of animals used (see Results section for additional details).

**Figure 5.**
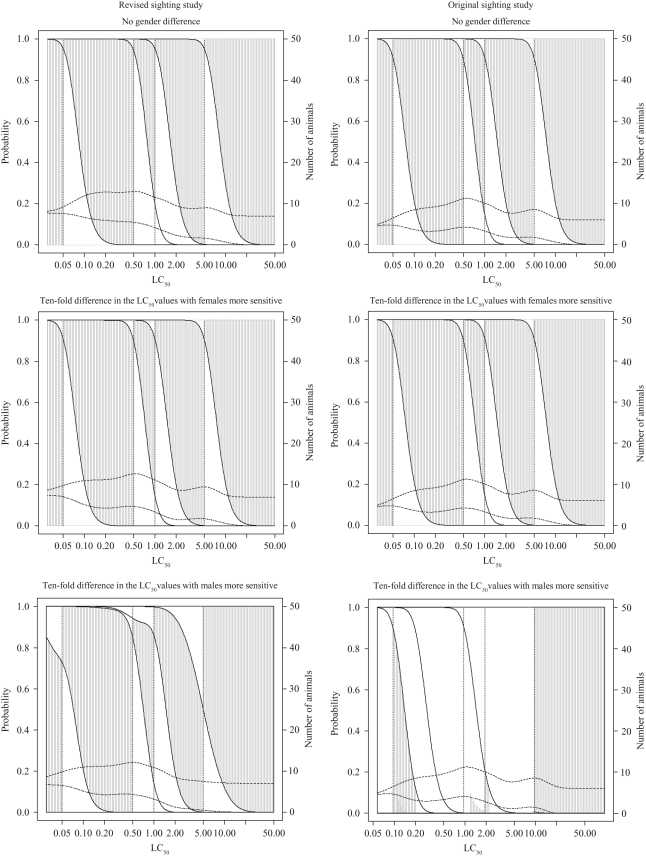
Classification probabilities and expected numbers of animals and deaths for the fixed concentration procedure (FCP) with the new sighting study for dusts and mists with concentration-response curve slope of 4 and *R* (LC_50_/TC_50_) of 5 assuming sighting study starting at 5 mg/L (see legend to Figure 4 and Results section text for additional details).

The results of the statistical evaluations for the three test procedures are summarized in Figures 4 and 5 and Tables 1–3. Figures 4 and 5 show some of the properties of the procedure for hypothetical dusts and mists with LC_50_ values ranging from 0.01 to 50 mg/L, *R* equal to 5 and concentration-response curve slope values of 4. To assess the effect of the sighting study starting concentration, the sighting study was assumed to start at either 0.05 mg/L or 5 mg/L, i.e. at the highest or lowest test concentration. In each case, results were obtained assuming no gender difference or a 10-fold difference in LC_50_ values for males and females. For comparison purposes, the right-hand columns of Figures 4 and 5 include plots that show the properties of the FCP with the original single gender sighting study.

**Table 1.** Classification probabilities and expected numbers of animals and deaths for the fixed concentration procedure (FCP) with the new sighting study for dusts and mists assuming no gender difference (see text for more details)

Substance		Classification probabilities					Mean no. animals	
LC_50_	Slope	Class 1	Class 2	Class 3	Class 4	Class 5	Tested	Deaths
0.03	4.0	**100.0**	0.0	0.0	0.0	0.0	2.2	1.8
0.15	4.0	6.3	**93.7**	0.0	0.0	0.0	6.8	0.3
0.70	4.0	0.0	69.1	**30.9**	0.0	0.0	10.2	1.3
1.00	4.0	0.0	29.6	**70.3**	0.0	0.0	7.6	0.7
1.10	4.0	0.0	21.5	78.5	**0.1**	0.0	7.4	0.5
2.50	4.0	0.0	0.0	13.3	**86.7**	0.0	7.3	0.4
10.00	4.0	0.0	0.0	0.0	29.6	**70.4**	7.6	0.7
0.03	10.0	**100.0**	0.0	0.0	0.0	0.0	2.0	2.0
0.15	10.0	0.0	**100.0**	0.0	0.0	0.0	7.0	0.0
0.70	10.0	0.0	17.7	**82.3**	0.0	0.0	9.2	0.5
1.00	10.0	0.0	0.3	**99.7**	0.0	0.0	7.0	0.0
1.10	10.0	0.0	0.1	99.9	**0.0**	0.0	7.0	0.0
2.50	10.0	0.0	0.0	0.0	**100.0**	0.0	7.0	0.0
10.00	10.0	0.0	0.0	0.0	0.3	**99.7**	7.0	0.0

**Table 2.** Classification probabilities and expected numbers of animals and deaths for the fixed concentration procedure (FCP) with the new sighting study for dusts and mists assuming females are more sensitive (see text for more details)

LC_50_ for males ten times greater than for females									
Substance			Classification probabilities					Mean no. animals	
LC_50_ (females)	LC_50_ (males)	Slope	Class 1	Class 2	Class 3	Class 4	Class 5	Tested	Deaths
0.03	0.3	4.0	**99.9**	0.1	0.0	0.0	0.0	2.9	1.6
0.15	1.5	4.0	3.5	**96.4**	0.0	0.0	0.0	7.3	0.4
0.70	7.0	4.0	0.0	58.6	**41.3**	0.0	0.0	10.6	1.3
1.00	10.0	4.0	0.0	20.5	**78.9**	0.5	0.0	7.8	0.7
1.10	11.0	4.0	0.0	14.1	84.7	**1.2**	0.0	7.7	0.6
2.50	25.0	4.0	0.0	0.0	8.2	**91.8**	0.0	7.5	0.5
10.00	100.0	4.0	0.0	0.0	0.0	20.6	**79.4**	7.6	0.6
0.03	0.3	10.0	**100.0**	0.0	0.0	0.0	0.0	2.1	1.1
0.15	1.5	10.0	0.0	**100.0**	0.0	0.0	0.0	7.0	0.0
0.70	7.0	10.0	0.0	11.3	**88.7**	0.0	0.0	9.2	0.4
1.00	10.0	10.0	0.0	0.1	**99.9**	0.0	0.0	7.0	0.0
1.10	11.0	10.0	0.0	0.0	100.0	**0.0**	0.0	7.0	0.0
2.50	25.0	10.0	0.0	0.0	0.0	**100.0**	0.0	7.0	0.0
10.00	100.0	10.0	0.0	0.0	0.0	0.1	**99.9**	7.0	0.0

**Table 3.** Classification probabilities and expected numbers of animals and deaths for the fixed concentration procedure (FCP) with the new sighting study for dusts and mists assuming males are more sensitive (see text for more details)

LC_50_ for females ten times greater than for males									
Substance			Classification probabilities					Mean no. animals	
LC_50_ (females)	LC_50_ (males)	Slope	Class 1	Class 2	Class 3	Class 4	Class 5	Tested	Deaths
0.3	0.03	4.0	**92.9**	7.1	0.0	0.0	0.0	3.0	1.3
1.5	0.15	4.0	3.5	**96.1**	0.4	0.0	0.0	7.2	0.4
7.0	0.70	4.0	0.0	57.5	**40.3**	2.0	0.2	10.7	1.3
10.0	1.00	4.0	0.0	20.4	**78.1**	1.0	0.5	7.8	0.7
11.0	1.10	4.0	0.0	14.0	84.0	**1.4**	0.5	7.7	0.6
25.0	2.50	4.0	0.0	0.0	8.1	**90.9**	0.9	7.5	0.4
100.0	10.00	4.0	0.0	0.0	0.0	19.9	**80.1**	7.5	0.6
0.3	0.03	10.0	**99.7**	0.3	0.0	0.0	0.0	2.1	1.0
1.5	0.15	10.0	0.0	**100.0**	0.0	0.0	0.0	7.0	0.0
7.0	0.70	10.0	0.0	11.3	**88.7**	0.0	0.0	9.2	0.4
10.0	1.00	10.0	0.0	0.1	**99.9**	0.0	0.0	7.0	0.0
11.0	1.10	10.0	0.0	0.0	100.0	**0.0**	0.0	7.0	0.0
25.0	2.50	10.0	0.0	0.0	0.0	**100.0**	0.0	7.0	0.0
100.0	10.00	10.0	0.0	0.0	0.0	0.1	**99.9**	7.0	0.0

For each LC_50_ value (plotted across the bottom of the graph), the first vertically sloping line shows the probability (according to the scale on the left-hand axis) of classification into class 1, the second into class 1 or 2 (so that the difference this and the one below is the probability of classification into class 2), the third into class 1, 2 or 3 (so that the difference between this and the one below is the probability of classification into class 3) and so on. The vertical dotted lines give the correct classes based on the true LC_50_ value and the dashed lines horizontally across the plot show the expected number of animals and deaths (using the scale on the right-hand axis, with the higher line representing the number of animals). For each LC_50_ value, the height of the shaded areas gives the probability of correct classification, the height of the area below the shaded area is the probability of classification into too toxic a class (impossible for true class 1) and the height of the area above the shaded area is the probability of classification into a class that is not toxic enough (impossible for true class 5). It should be noted in the interpretation of the figures that true LC_50_ values are not evenly spread across the range illustrated. In particular, substances with higher LC_50_ values are much more common than those with the lower values. Properties of the procedures for the majority substances are thus given by the curves towards the right-hand side of the plots, although classification of more toxic substances remains important.

Tables 1 to 3 give the properties of the procedure for hypothetical dusts and mists with LC_50_ values 0.03, 0.15, 0.7, 1, 1.1, 2.5 and 10 mg/L, *R* equal to 5 and concentration-response curve slope values of 4 and 10. The sighting study starting concentration was assumed to depend on the true LC_50_ to reflect the situation in which prior knowledge of the test substance is used to choose the initial concentration. As such, for the hypothetical substances listed above, starting concentrations of 0.05, 0.05, 0.05, 0.5, 0.5, 1 and 5 mg/L were used, respectively. In practice, if prior knowledge was available to determine the sighting study starting concentration, a sighting study may not be needed and the procedure could commence with the main study. However, this would depend on the reliability of the prior information. As in the figures, results in the tables are presented assuming no gender difference or a 10-fold difference in LC_50_ values for males and females. The probabilities of classification into the correct GHS class based on the true LC_50_ value are shown in bold.

In the absence of a gender difference, the new procedure is slightly more stringent than the FCP using the original single-gender sighting study.^5^ For small gender differences, the performance of the procedure will be similar to that when there is no gender difference. However, as the difference between the genders increases, an observation of death or non-fatal toxicity in one of the genders drives the choice of starting concentration, and it is easier to identify the more sensitive gender for subsequent use in the main study. As such, in the presence of larger gender differences, the revised sighting study substantially improves the performance of the FCP.

The default for the FCP main study, which remains unchanged, is to use females. This means that the FCP with the revised sighting study is most like the FCP with the original single-gender sighting study when females are the more sensitive gender. In fact, a comparison of the probabilities in Table 2 (females more sensitive) with those generated by Price et al.^5^ for the case of no gender difference using the original sighting study shows nearly identical probabilities of correct classification.

If males are more sensitive than females and the concentration-response curve is shallow, in this case a slope of 4, there is a small chance that evident toxicity or death will occur at the same concentration in both males and females. In this case, the main study would use females despite them being the less sensitive gender. The result of this is that the procedure is a little less stringent when males are more sensitive than females, with a slightly higher chance of misclassification into a less toxic class. As shown in Table 3, this is particularly true for substances with a true LC_50_ of 0.03 mg/L belonging to the most toxic class 1. In this case, there is a 7% chance of misclassification into the less toxic class 2 when males are more sensitive than females compared to a 0.1% chance when females are more sensitive than males (Table 2). It should be stressed, however, that the chance of under-classification is small and certainly no larger than for other test procedures. The substantial under-classification observed for the original female-only FCP^5^ when females are less sensitive than males is avoided.

The number of animals required in the FCP with revised sighting study is, not surprisingly, slightly higher than for the FCP using the original sighting study, since the revised sighting study requires exposure of both males and females. When the sighting study starts at a concentration above the LC_50_, the number of deaths is also increased slightly. Despite this, the number of animals exposed and the number of deaths remain considerably lower than for other test procedures.

## Discussion

In this paper, we have proposed a new sighting study for the FCP. In the original sighting study, a single female animal is tested at each concentration in order to determine an appropriate starting concentration for the main study. The main study is then conducted using females. In the revised sighting study, two animals, one male and one female, are tested at each concentration. If a gender difference is apparent, the main study is conducted in the more sensitive gender, otherwise females are used.

This modification is proposed in light of our previous analyses that demonstrated the potential for gender differences in sensitivity to acute inhalation toxicity, with males or females being the more sensitive gender, and the impaired performance of the FCP when males are more sensitive.^5^ In the absence of a gender difference, the classification performance of the FCP is good and is broadly comparable to both the LC_50_ method (OECD TG 403^1^) and the ATC method (OECD TG 436^2^), which are currently used for the assessment of acute inhalation toxicity.

A statistical evaluation of the new procedure has been reported here. It is shown that if there is no gender difference in sensitivity to acute inhalation toxicity, the new procedure is slightly more stringent than the original. This is unsurprising since the main procedure is more likely to start at a lower concentration. To illustrate this, suppose that evident toxicity is possible at 1 mg/L but very unlikely at any lower concentration. According to the original sighting study, if toxicity is observed for the single animal tested at 1 mg/L, the main study will start at 1 mg/L. If toxicity is not observed, the main study will start at 5 mg/L. With the modified sighting study, the main study will start at 1 mg/L if either of the two animals tested at 1 mg/L demonstrate toxicity, and at 5 mg/L otherwise. The probability of observing signs of toxicity in at least one animal is obviously higher when two animals are observed than when one is observed, so that the lower starting concentration is more likely for the revised sighting study than for the original. This in turn leads to an increased chance of observing evident toxicity or death at the lower concentration, and hence to a more stringent classification.

If females are more sensitive than males, the classification properties of the new procedure are nearly identical to those of the original FCP, which uses females by default. Moreover, the larger the gender difference, the more similar the procedures become due to the increased likelihood of selecting females for the main study.

If males are more sensitive than females, the original procedure, which does not generally test males, carries a high risk of under-classifying substances. This was a cause for concern given that males have been shown to be more sensitive than females to some substances, and that this might not be anticipated prior to testing. While the new procedure, which includes males in the sighting study, does not completely eradicate under-classification, it corrects the tendency of the original FCP to substantially under-classify substances when males are more sensitive than females.

As in previous similar statistical evaluations, the results are based on the assumption that concentration-response curves are of the probit form. An additional assumption is that these curves have equal slopes for males and for females and for lethality and toxicity. Whilst further evaluations could be conducted based on different statistical modelling assumptions, the qualitative comparison of the procedures is unlikely to be changed.

In all cases, the new procedure uses a slightly larger number of animals than the original FCP, since pairs of animals rather than single females are now required in the sighting study. However, animal numbers remain considerably lower than for other test methods, and the test continues to provide a refinement. We therefore consider that this additional cost is worthwhile in light of the improved characteristics of the new procedure.
